# Metastatic Breast Cancer Coexisting With HER-2 Amplification and EGFR Exon 19 Deletion Benefits From EGFR-TKI Therapy: A Case Report

**DOI:** 10.3389/fonc.2020.00771

**Published:** 2020-05-29

**Authors:** Wei Jing, Jie-Tao Ma, Cheng-Bo Han

**Affiliations:** Department of Clinical Oncology, Shengjing Hospital of China Medical University, Shenyang, China

**Keywords:** breast cancer, lung metastasis, EGFR mutation, HER-2 amplification, molecular targeted therapy

## Abstract

**Background:** Patients with different molecular subtypes of breast cancers have different recurrence risks and prognoses. Clinical support and evidence to guide management are absent for patients with breast cancer coexisting with HER-2 amplification and EGFR mutations.

**Case presentation:** We report a case of breast cancer coexisting with HER-2 amplification and EGFR exon 19 deletion (E19 del). The patient presented with solitary pulmonary nodule and enlargement of hilar and mediastinal lymph nodes 2 years after radical mastectomy. Biopsy of the subcarinal lymph node showed suspected adenocarcinoma. The specimen was too small for further immunohistochemistry, but an EGFR E19 del was discovered. Due to the primary diagnosis of EGFR-mutant lung adenocarcinoma, EGFR-TKI gefitinib was administered and resulted in 1 year of stable disease until the patient developed progression in the right pulmonary nodule with new metastatic cervical lymph nodes. According to histopathological findings of re-biopsy of the pulmonary nodule and left cervical and subcarinal lymph nodes, the patient was diagnosed with breast cancer with lung metastasis and multiple lymph node metastases. The patient received multiple anti-HER-2-targeted therapies (trastuzumab for 9.7 months, lapatinib for 9 months, and pyrotinib for 4+ months) and survived for more than 36 months after lung metastasis.

**Conclusions:** This case suggested that breast cancer coexisting with HER-2 amplification and EGFR E19 del may be driven by both HER-2 and EGFR signaling pathways, and patients can benefit from EGFR-TKI and anti-HER-2 therapy.

## Introduction

Breast cancer, as the most common cancer in females, is reported to have had about 252,710 new cases in the United States during 2017, accounting for 30% of all new cancer diagnoses in women (1). Most breast cancer patients need different sets of adjuvant or neoadjuvant therapies based on staging, molecular typing, and risk factors for recurrence, including chemotherapy, endocrine therapy, targeted therapy, and radiation therapy (2). With the development of therapeutic drugs and novel drug-delivery vehicles, especially targeted drugs and chemotherapy medicine delivered by nanoparticles, the mortality of breast cancer has declined over the past two decades. Patients with either locally advanced and metastatic breast cancer have notably longer overall survival (3). Nevertheless, two peaks of relapse may occur during the first 5 years after surgery for general breast cancer patients. The lung is one of the most common metastatic sites in patients with advanced breast cancer. Nearly 60% to 70% of patients with metastatic breast cancer die with lung metastasis (4). Patients with lung metastasis have a median overall survival of 22 months (5).

High heterogeneity is a hallmark of breast cancer. Patients with breast cancers with different molecular subtypes have different recurrence risk and survival prognosis. About 20–25% of breast cancer patients have HER-2 protein overexpression or gene amplification, which is considered an effective predictor for anti-HER-2 therapy (6). Reports indicated that 2 of 139 (1.4%) patients with breast cancer harbored EGFR mutations but EGFR mutations occurred in nearly 11.4% of triple negative breast cancer (TNBC) patients (7, 8). The frequency of EGFR mutations has not been reported in HER-2-positive breast cancer. Here, we report a case of breast cancer with atypical lung metastasis coexisting with HER-2 amplification and epidermal growth factor receptor (EGFR) exon 19 deletion (E19 del). The patient was misdiagnosed with metachronous HER-2-amplified breast cancer and EGFR-mutant lung adenocarcinoma. The patient benefited from EGFR-TKI therapy.

To our knowledge, this may be the first case report to discuss the efficacy of EGFR-TKIs as first-line therapy for HER-2-positive advanced breast cancer with EGFR co-mutation. Therefore, we summarized the diagnosis and treatment of this patient and discussed the experience and lessons learned from it.

## Case Presentation

### Surgery and Adjuvant Therapy

A 50-year-old female patient was clinically diagnosed with left breast cancer and received a radical mastectomy in February 2014. Pathological findings supported invasive ductal carcinoma with immunohistochemistry (IHC) for ER 80%+, PR 70%+, Ki-67 20%+, and fluorescence *in situ* hybridization (FISH) for HER-2 amplification. The patient had early stage disease (pT1N0M0, stage I) and underwent 8 cycles of adjuvant chemotherapy (CE ×4 → *T*×4) without trastuzumab followed by tamoxifen.

### EGFR-TKI Therapy

**Figure 1:**
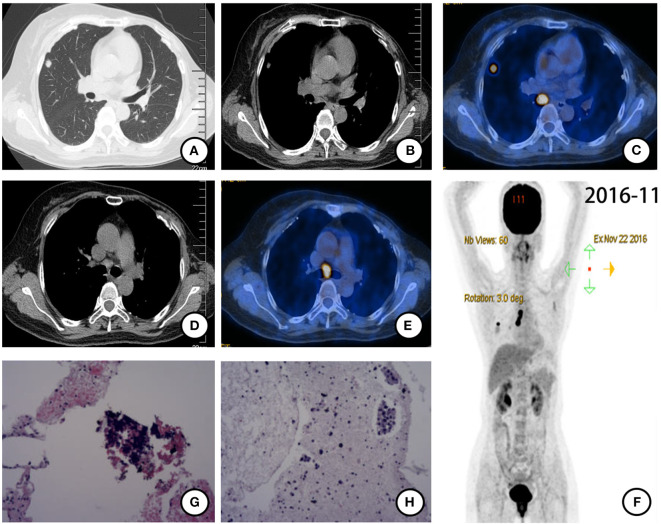
PET-CT scan (November 2016) and histopathological results after postoperative recurrence. **(A)** Solitary right pulmonary nodule and **(B)** enlarged right hilar lymph node were found and identified as metabolically active lesions (SUVmax = 8.32) **(C)**. **(D,E)** Subcarinal lymph node enlarged with intense FDG uptake (SUVmax = 13.19). **(F)** Holistic view of PET-CT: metabolic lesions in the lung, hilum, and subcarina. **(G)** Histopathological features of CT-guided biopsy tissue of the nodule in the right upper lobe: alveolus tissue with some naked nuclear-like lymphocytes. (×20 hematoxylin/eosin). **(H)** Histopathological features of EBUS biopsy tissue of the subcarinal lymph node: cancer cells arranged in disperse or the nest bulk in mucoid tissue (×20 hematoxylin/eosin).

Follow-up chest computed tomography (CT) in November 2016 showed a nodule in the upper lobe of the right lung. The patient complained of chest pain but denied fever, cough, hemoptysis, and weight loss. She had no family history of cancer. She had menarche at 13 and was not currently in menopause. Further positron emission tomography CT (PET-CT) showed a lobulated nodule with diameter 0.8 ×0.9 cm in the upper lobe of the right lung with intense fluorodeoxyglucose (FDG) uptake (SUVmax = 8.32) (Figures 1A–C). The enlarged right hilar and subcarinal lymph nodes were also identified as metabolically active lesions (SUVmax = 13.19) (Figures 1D,E). There were no signs of other distant metastasis (Figure 1F). CT-guided biopsy of the nodule in the right upper lobe was performed for histopathological diagnosis. Pathologic assessment of the biopsy tissue revealed alveolus tissue (Figure 1G), with positive expression for LCA but negative expression for CK, CD56, Syn, and TTF-1. Further histopathological diagnosis was suspected adenocarcinoma after endobronchial ultrasound (EBUS) biopsy of the subcarinal lymph node (Figure 1H). The specimen was too small for further immunohistochemistry (IHC). An EGFR E19 del was discovered by the ADx-ARMS method (Supplementary Figure 1).

Based on the pathological and molecular results and CT findings, a primary lung adenocarcinoma was suspected. Since radical surgery was not available, the patient started receiving EGFR-TKI gefitinib (250 mg once daily) and stopped taking tamoxifen since November 2016. Follow-up chest CT indicated stable disease until November 2017, when the patient presented left neck swelling at physical examination.

### Anti-HER-2 Therapy

**Figure 2:**
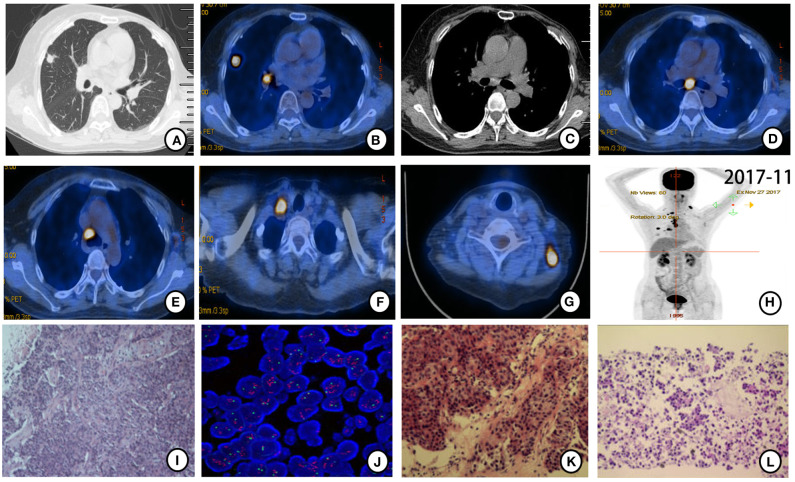
PET-CT scan (November 2017) and histopathological and molecular results after gefitinib therapy resistance. **(A,B)** Original mass in the upper lobe of the right lung enlarged (1.4 ×1.1 cm) with more intense FDG uptake (SUVmax = 11.24). **(C,D)** Right hilar and subcarinal lymph node enlarged with more intense FDG uptake. **(E–G)** New mediastinal, right subclavian, and bilateral cervical lymph nodes were found with FDG uptake. **(H)** Holistic view of PET-CT: metabolic lesions in multiple metastases. Histopathological features of ultrasound-guided biopsy tissue. **(I)** Cancer cells arranged in streaks and nest bulk and invasive growth manner in left cervical lymph nodes (×20 hematoxylin/eosin). **(J)** HER-2 amplification was detected by FISH in left cervical lymph nodes tissue. HER-2 signal (red) was found in clusters distributed. **(K)** Irregular adenoid and cord-like cancer cells were found in the biopsy tissue from the nodule in the upper lobe of the right lung (×20 hematoxylin/eosin). **(L)** Cancer cells scattered or arranged in groups observed in right hilar and subcarinal lymph nodes tissues acquired by EBUS-guided biopsy (×20 hematoxylin/eosin).

Further PET-CT in November 2017 revealed more intense FDG uptake in the right lung nodule (1.4 ×1.1 cm, SUVmax = 11.24) and hilar and subcarinal lymph nodes (Figures 2A–E). Enlargement in bilateral cervical and right subclavian lymph nodes was found (Figures 2F–H). Ultrasound-guided biopsy of left cervical lymph nodes was performed for histopathological diagnosis. HE staining (Figure 2I) combined with IHC (positive expression for ER [80%], PR [20%], HER-2 [2–3+], Gata-3, GCDFP-15 and mammaglobin, but negative expression for TTF-1 and Napsin A), and FISH (HER-2 amplification, Figure 2J) suggested that metastatic cervical lymph nodes derived from breast cancer. CT-guided biopsy of the nodule in the upper lobe of the right lung and EBUS-guided biopsy of the right hilar and subcarinal lymph nodes were also performed for further histopathological diagnosis. Irregular adenoid and cord-like cancer cells were found in the biopsy tissue from the nodule in the upper lobe of the right lung (Figure 2K) with IHC results of positive expression for ER (80%), PR (20%), HER-2 (2-3+), Ki-67 (10%), and Gata-3, but negative expression for TTF-1, Napsin A, and P40 (Supplementary Figure 2). Cancer cells scattered or arranged in groups were observed in biopsy tissues of right hilar and subcarinal lymph nodes (Figure 2L) with IHC results of positive expression for ER (80%), PR (20%). HER-2 (2–3+), CK, CK7, mammaglobin and Gata-3, but negative expression for TTF-1, and Napsin A. HE staining (Figures 2K,L) combined with IHC (positive expression for ER [80%], PR [20%], and HER-2 [3+], but negative expression for TTF-1) confirmed all the biopsy sites metastasized from breast cancer.

**Figure 3:**
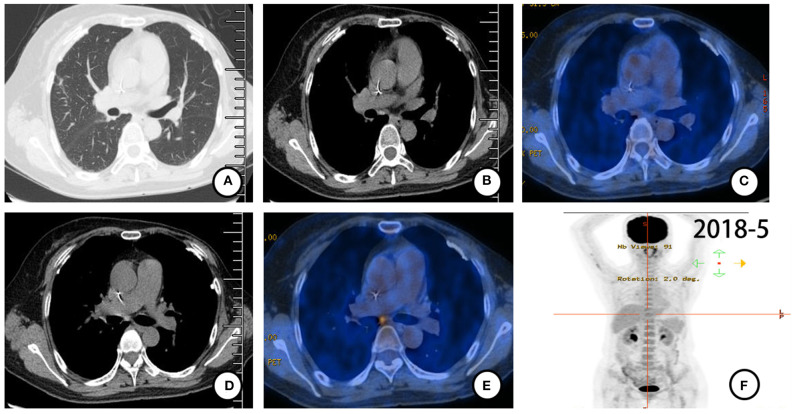
PET-CT (May 2018) scan after 6 cycles of combination treatment with trastuzumab, vinorelbine, and capecitabine. **(A–F)** All metastases shrank with almost no FDG uptake.

**Figure 4:**
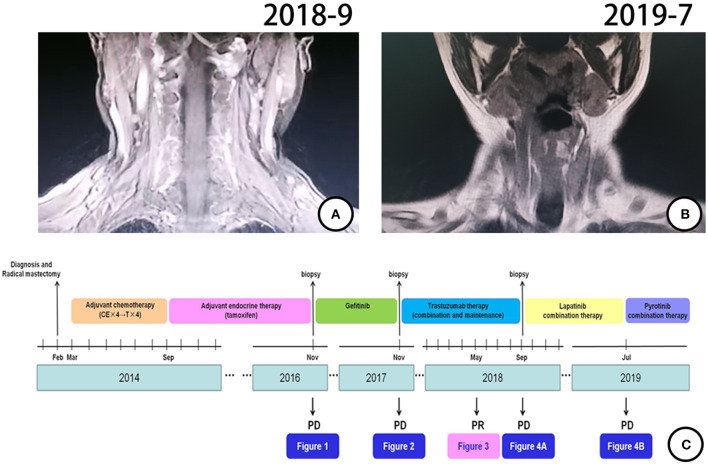
Treatment process and effect evaluation of the patient. **(A)** New metastatic lymph node in left neck confirmed by MR in September 2018. **(B)** New metastatic lymph node of right neck confirmed by MR in July 2019. **(C)** Review of treatment process (from February 2014 to November 2019).

According to pathological findings, primary lung adenocarcinoma was excluded. The patient had breast cancer with lung metastasis and multiple lymph node metastases. She received 6 cycles of combination treatment with trastuzumab, vinorelbine, and capecitabine from December 2017. Repeated PET-CT in May 2018 indicated that all metastatic lesions significantly shrank (Figure 3). Thereafter, the patient continued on trastuzumab and capecitabine every 3 weeks as maintenance therapy. After 9.7 months of treatment with trastuzumab, the patient was pathologically confirmed as having a new metastatic lymph node in the left neck and received capecitabine combined with lapatinib from September 2018 (Figure 4A). The patient had stable disease for over 9 months until a new progressive disease was found in the right cervical lymph node in July 2019 (Figure 4B). She was then treated with pyrotinib, a novel irreversible EGFR/HER-2 dual tyrosine kinase inhibitor, and achieved stable disease for more than 4 months by November 2019. The patient survived for 36 months after lung metastasis and 69 months after diagnosis of breast cancer (Figure 4C).

## Discussion

In this case report, a patient had HER-2-amplified and ER-expressing breast cancer. The patient received a radical mastectomy, postoperative adjuvant chemotherapy, and endocrine therapy without anti-HER-2-targeted therapy. Metachronous thoracic metastases occurred 33 months after mastectomy, with a solitary nodule in the right upper lobe and right hilar and subcarinal metastatic lymph nodes. The primary diagnosis of EGFR-mutant lung adenocarcinoma was made based on the insufficient immunohistochemistry and atypical CT appearance of lung metastasis. The first-line administration of EGFR-TKI gefitinib unexpectedly resulted in 1 year of stable disease. The patient benefited from both EGFR-TKI and anti-HER-2 therapy and survived more than 3 years after lung metastasis.

Currently, subtype classification based on molecular biomarkers is recommended to guide breast cancer treatment. In addition, multigene panel testing is deemed a useful supplement for deciding on postoperative adjuvant therapy for patients with early breast cancer (9). Different subtypes may have distinctive organ metastasis ability and prognosis (10). HER-2 gene amplification causes overexpression of HER-2 protein, which induces the homologous or heterogeneous dimerization of HER-2 receptors and subsequent activation of HER-2 downstream MEK, AKT, and PKC signaling pathways, driving proliferative, invasive, and metabolic processes (11). Patients with HER-2-amplified breast cancer with lung metastasis have worse prognosis than patients with hormone receptor-positive subtypes (12). Tumor heterogeneity is an important biological characteristic of tumors and is closely related to treatment effect. Tissue biopsy at specific time points and detection of related genes and proteins can help clinicians judge genetic heterogeneity, which can guide subsequent therapy. In our case, the patient refused anti-HER-2 adjuvant therapy, increasing the recurrence risk. Trastuzumab combined with chemotherapy for this patient obtained a PFS of over 9 months, similar to the results of clinical trials (13). Besides the fact that the EGFR E19 del was discovered at the first relapse, the status of the HER-2 gene in metastatic cervical lymph nodes was confirmed as amplification, which verified the initial diagnosis of metastatic breast cancer after resistance to gefitinib. We hypothesize that EGFR E19 del and HER-2 amplification originated from monoclonal or polyclonal tumor cells dominated by HER-2 amplification.

When an EGFR E19 del was detected, our patient received gefitinib treatment and benefited for nearly 12 months. Heterodimerization of EGFR and HER-2 triggers activation of downstream signaling and is considered an alternative mechanism of trastuzumab resistance. EGFR, as a member of the ERBB family, is dysregulated in many types of human carcinogenesis and cancer progression. EGFR overexpression, partly induced by gene amplification, is observed in 15–30% of breast cancers. It is considered an adverse clinical factor and a promising therapeutic target for breast cancer, especially triple-negative breast cancer (14, 15). EGFR E19 mutations occur at ATP-binding sites in the intracellular catalytic kinase domain, which induce autophosphorylation and constitutive activation of downstream EGFR pathways such as the MAPK, STAT3, and AKT pathways (16). E19 del and E21 L858R in EGFR are the most common driver mutations in non–small cell lung cancer (NSCLC). Multiple phase III clinical trials have demonstrated that EGFR-TKIs significantly prolong PFS compared with chemotherapy in patients with EGFR-mutant NSCLC (17). Gefitinib was found to inhibit growth of breast cancer cells with EGFR mutations *in vitro* (18). However, no significant clinical benefit from gefitinib was observed in breast cancer patients (19), even in TNBC and basal-like and inflammatory breast cancer with EGFR overexpression. We propose that the possible reasons include not considering the mutation status of EGFR and the lack of dependence on the EGFR pathway. Only the subgroup of patients with breast cancer with EGFR mutation, regardless of HER-2 status, may benefit from EGFR-TKI therapy. Other patients with HER-2-positive (EGFR-independent) disease hardly benefit from EGFR-TKI treatment (20). Several reports suggest that HER-2 signaling contributes to EGFR-TKI resistance in patients with EGFR-mutant disease. HER-2 amplification occurs in approximately 10%−15% of EGFR-mutant lung cancers with acquired resistance to EGFR-TKIs (21). *In vitro*, acquired resistance to EGFR-TKIs mediated by activation of HER-2 can be overcome by inhibition of HER-2 (22). HER-2-targeted therapy is now a promising treatment strategy to overcome HER-2-dependent resistance to EGFR-TKIs (23). The case we present suggested that EGFR mutations play a driver role, even when accompanied by HER-2 amplification. Therefore, we hypothesize that the subset of breast cancers with EGFR mutations might respond to EGFR-TKI therapy. However, the underlying molecular mechanisms need to be further studied.

The patient was misdiagnosed as having local advanced lung adenocarcinoma due to typical imaging features of primary lung cancer and an EGFR mutation in the adenocarcinoma of the mediastinal lymph nodes. Usually, the diagnosis of lung metastasis mainly depends on previous tumor history and radiological appearance. The typical CT appearance of lung metastases is mostly multiple nodules scattered in both lungs. Solitary pulmonary nodules and enlargement of pulmonary hilar or mediastinal lymph nodes are extremely rare in metastatic breast cancer. In our case, the patient presented only with solitary right pulmonary nodule and right hilar and subcarinal lymphadenopathy 2 years after radical mastectomy for breast cancer. The tissue specimen was too small for IHC confirmation. Based on the interval for radical mastectomy, the typical and similar PET-CT appearance of primary lung cancer, and the molecular and pathological findings of adenocarcinoma with EGFR E19 del mutation, primary lung adenocarcinoma diagnosis was made at first relapse (November 2016). One year later, new metastases in the left cervical lymph nodes were found and biopsied for histopathological diagnosis. HE staining, IHC (positive expression for ER [80%], PR [20%], Gata-3, GCDFP-15, and mammaglobin, but negative expression for TTF-1 and Napsin A) and FISH (HER-2 amplification) suggested metastatic cervical lymph nodes derived from breast cancer. A second CT-guided biopsy of the nodule in the upper lobe of the right lung was performed. Irregular adenoid and cord-like cancer cells were found in the biopsy tissue of the nodule in the upper lobe of the right lung with IHC results of positive expression for ER (80%), PR (20%), HER-2 (2–3+), Ki-67 (10%), and Gata-3, but negative expression for TTF-1, Napsin A, and P40. This result helped to confirm the diagnosis of lung metastasis of breast cancer after EGFR-TKI therapy failure in November 2017. Limited results from pathologic diagnosis and atypical imaging of lung metastasis led to the misdiagnosis. Pathology is considered an indispensable diagnostic tool for differential diagnosis. Histopathological and immunohistochemical analyses and genetic testing of biopsy tissues are crucial for accurate diagnoses, especially when patients have atypical metastatic tumor imaging.

## Conclusion

This case suggested that breast cancer coexisting with HER-2 amplification and EGFR E19 del may be driven by both signaling pathways and patients can benefit from EGFR-TKIs and anti-HER-2 therapy. Early identification and accurate diagnosis are not only fundamental principles of cancer therapy but also crucial for determining therapeutic schemes and prognostic survival.

## Ethics Statement

Written informed consent was obtained from the patient for publication of this case report and accompanying images. This report adhered to the tenets of the Declaration of Helsinki and was reviewed and approved by our center's ethics committee (Shengjing Hospital of China Medical University, Shenyang, China).

## Author Contributions

WJ was mainly responsible for the article writing. C-BH was the corresponding author. WJ and J-TM were responsible for patients' clinical data and analysis.

## Conflict of Interest

The authors declare that the research was conducted in the absence of any commercial or financial relationships that could be construed as a potential conflict of interest.

## Supplementary Material

Supplementary Figure 1 Deletion in EGFR at exon 19.

Supplementary Figure 2 Immunohistochemical expression in biopsy tissue from the nodule in the right lung upper lobe after a second CT-guided biopsy (November 2017). Representative IHC image for **(A)** ER (80% positive), **(B)** PR (20% positive), **(C)** HER-2 (2-3 positive), **(D**) Ki-67 (10% positive), **(E)** Gata-3 (positive), **(F)** TTF-1 (negative), **(G)** Napsin A (negative), and **(H)** P40 (negative).
