# Supplementary figures and images for: Metastatic Breast Cancer Coexisting With HER-2 Amplification and EGFR Exon 19 Deletion Benefits From EGFR-TKI Therapy: A Case Report
Source: Front Oncol. 2020 May 29;10:771. doi: 10.3389/fonc.2020.00771 (PMC7274020; doi:10.3389/fonc.2020.00771)

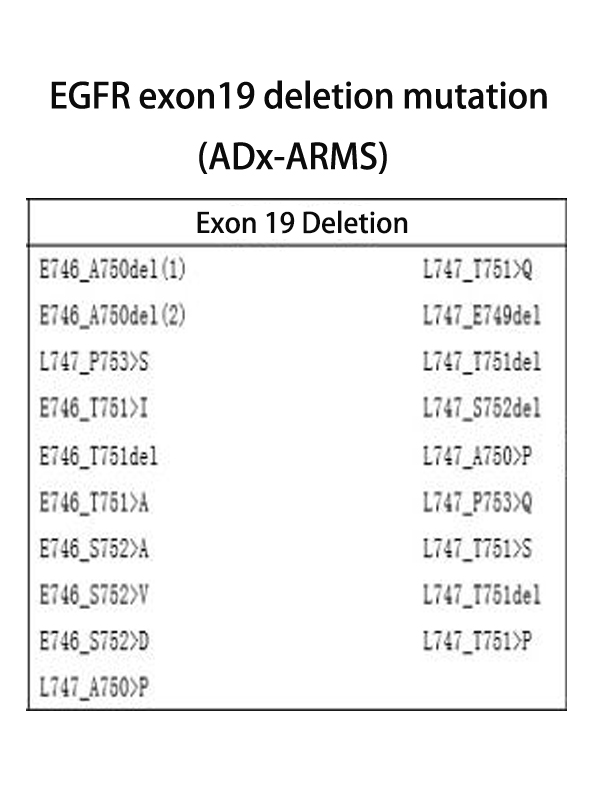

Supplement: Supplementary Figure 1 — Deletion in EGFR at exon 19. [file Image_1.JPEG]

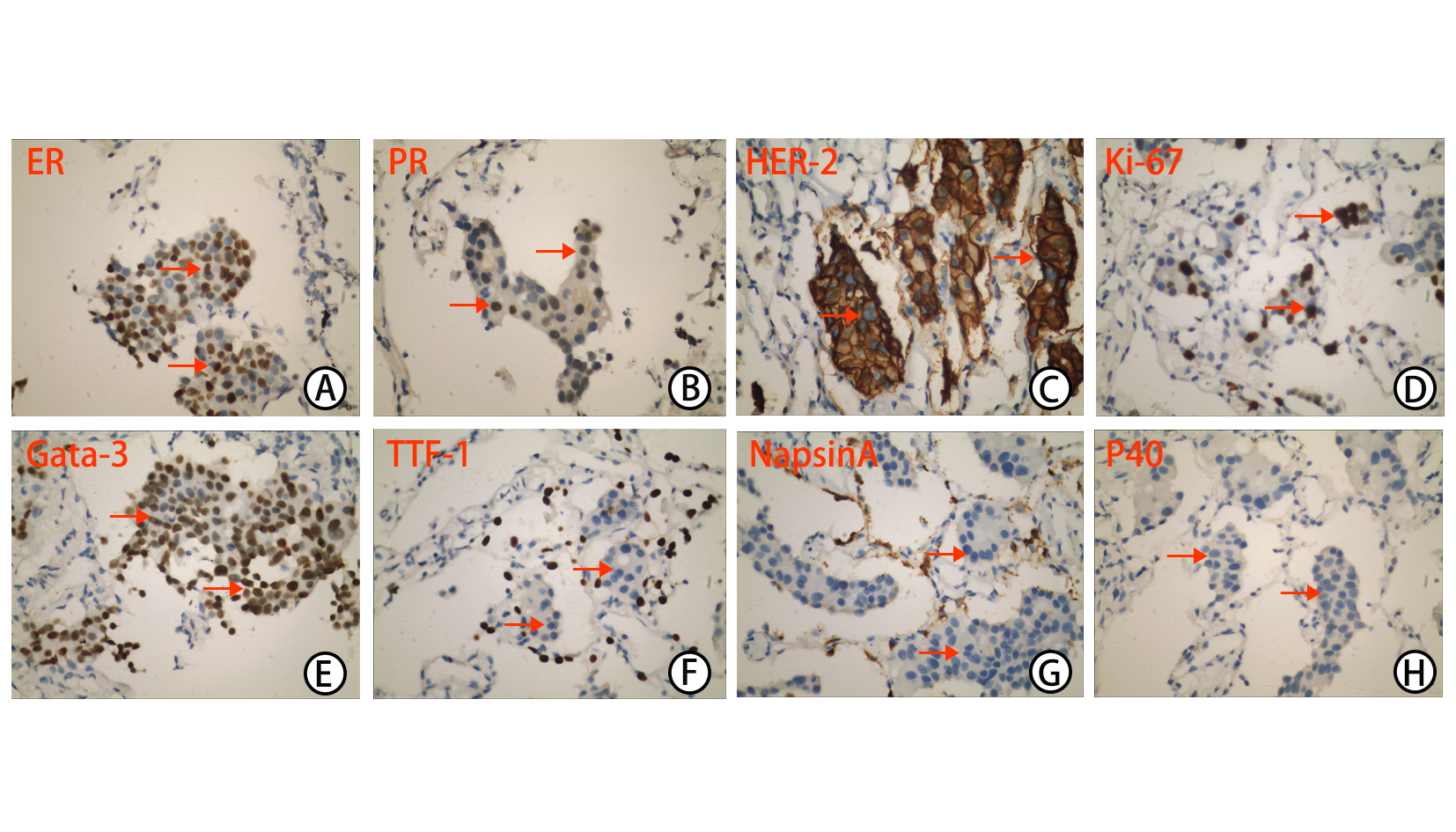

Supplement: Supplementary Figure 2 — Immunohistochemical expression in biopsy tissue from the nodule in the right lung upper lobe after a second CT-guided biopsy (November 2017). Representative IHC image for (A) ER (80% positive), (B) PR (20% positive), (C) HER-2 (2-3 positive), (D) Ki-67 (10% positive), (E) Gata-3 (positive), (F) TTF-1 (negative), (G) Napsin A (negative), and (H) P40 (negative). [file Image_2.JPEG]
